# A psychometric analysis and revalidation of the Yale-Brown Obsessive Compulsive Scale modified for Binge Eating in adults with binge eating disorder

**DOI:** 10.1007/s11136-019-02277-8

**Published:** 2019-08-31

**Authors:** Karen Yee, Daniel Serrano, Judith Kando, Susan L. McElroy

**Affiliations:** 1 Shire, a member of the Takeda group of companies, Lexington, MA USA; 2 Pharmerit International, Bethesda, MD USA; 3 Lindner Center of HOPE, Mason, OH USA; 4 University of Cincinnati College of Medicine, Cincinnati, OH USA

**Keywords:** Binge eating disorder, Calibration, Psychometrics, Reliability, Yale-Brown Obsessive Compulsive Scale modified for Binge Eating

## Abstract

**Purpose:**

The Yale-Brown Obsessive Compulsive Scale modified for Binge Eating (Y-BOCS-BE) assesses the obsessiveness of binge eating thoughts and compulsiveness of binge eating behaviors. The findings of this study extend previously published Y-BOCS-BE psychometric evaluations in adults with binge eating disorder (BED).

**Methods:**

Data from three phase 3 lisdexamfetamine dimesylate studies in adults with BED (two randomized, double-blind, placebo-controlled short-term efficacy studies; one double-blind, placebo-controlled, randomized-withdrawal maintenance-of-efficacy study) were used. Psychometric evaluations included assessment of Y-BOCS-BE dimensionality, internal consistency, convergent validity, test?retest reliability, and determinations of clinically meaningful improvement using distribution- and anchor-based methods.

**Results:**

Domain specification analyses determined that the Y-BOCS-BE possessed a bifactor structure composed of a general binge eating severity domain and three subdomains (obsessive/compulsive, restraint, and control). Y-BOCS-BE internal consistency was maximized at week 12 (Cronbach?s *?*, 0.95) and test?retest reliability was maximized in the 8-week retest interval from week 4 to week 12 across all no-change anchors (*r*?=?0.74?0.90). Likewise, convergent validity of the Y-BOCS-BE across all validators was maximized at week 12 (all *r*???0.66). Meaningful improvement for Y-BOCS-BE total scores was estimated to require score reductions of 12 to 17 points depending on the anchor.

**Conclusions:**

The Y-BOCS-BE is a valuable tool for assessing BED symptoms. Maximization of Y-BOCS-BE reliability and validity at later study time points may be related to both treatment effects and improved insight into BED by participants during the study.

**Electronic supplementary material:**

The online version of this article (10.1007/s11136-019-02277-8) contains supplementary material, which is available to authorized users.

## Introduction

The Yale-Brown Obsessive Compulsive Scale modified for Binge Eating (Y-BOCS-BE), which assesses the obsessiveness of binge eating (BE) thoughts and compulsiveness of BE behaviors [1], is a modified version of the Yale-Brown Obsessive Compulsive Scale [2]. The Y-BOCS-BE has been used in studies of binge eating disorder (BED) to assess the efficacy of pharmacotherapy [3?^6^]. Across studies, reductions in BE were accompanied by statistically significant reductions in Y-BOCS-BE total score [3?^6^].

Psychometric testing and analysis of the Y-BOCS-BE is being conducted as a multi-stage process to optimize the characterization of BED. A preliminary validation of the Y-BOCS-BE [1] was conducted using data from a phase 2 study of lisdexamfetamine dimesylate (LDX) in adults with moderate-to-severe BED [7]. This analysis demonstrated that the Y-BOCS-BE had high internal consistency (Cronbach?s *?*, 0.81) at baseline [1]. The Y-BOCS-BE also exhibited good construct validity in relation to multiple reference measures, including the Three-Factor Eating Questionnaire (TFEQ) and Binge Eating Scale (BES), but correlations with reference measures at baseline were lower than for score changes from baseline to end-of-study [1]. The estimated range of Y-BOCS-BE total score reductions indicative of minimal clinically important change was 4 to 17 points [1]. These data suggested the Y-BOCS-BE is a reliable and valid measure of treatment benefit in BED [1].

This study extends the preliminary psychometric validation of the Y-BOCS-BE by using two phase 3 trials in adults with BED for validation and another phase 3 trial for characterization of treatment benefit on developed scores [6, 8]. LDX was superior to placebo in reducing BE days/week in the two short-term efficacy studies [6] and in prolonging time to relapse in the randomized-withdrawal maintenance-of-efficacy study [8]. The Y-BOCS-BE was included as a secondary efficacy endpoint in each of the studies. In the short-term efficacy studies, LDX was superior to placebo in reducing Y-BOCS-BE total score [6]. In the maintenance-of-efficacy study, LDX maintained Y-BOCS-BE total score reductions during the randomized-withdrawal phase [8]. These analyses further examine the dimensionality, item-level properties, scoring, and minimal clinically important improvement (MCII) of the Y-BOCS-BE and LDX treatment effects on Y-BOCS-BE scores from the aforementioned studies.

## Methods

### Study design and participants

Three multicenter LDX clinical studies, the designs of which have been described [6, 8], were used in these analyses. The short-term efficacy studies were identically designed, 12-week, randomized, double-blind, placebo-controlled studies (ClinicalTrials.gov, NCT01718483 [study 1] and NCT01718509 [study 2]) [6]. The maintenance-of-efficacy study was a double-blind, placebo-controlled, randomized-withdrawal study (ClinicalTrials.gov, NCT02009163 [study 3]), with a 12-week open-label phase and 26-week double-blind, randomized-withdrawal phase [8].

Comprehensive inclusion and exclusion criteria have been published [6, 8]. In summary, all studies enrolled men and nonpregnant women (age, 18?55 years; body mass index [BMI], ??18 to ??45 kg/m^2^). All participants met *Diagnostic and Statistical Manual of Mental Disorders, Fourth Edition, Text Revision*, BED criteria and had protocol-defined moderate-to-severe BED (??3 BE days/week for 14 days before baseline and Clinical Global Impressions-Severity [CGI-S] ratings ??4 at screening and baseline). Key exclusion criteria included current anorexia nervosa or bulimia nervosa diagnoses; current comorbid Axis I or Axis II psychiatric disorders controlled with prohibited medications or uncontrolled and associated with significant symptoms; a history of cardiovascular health problems; clinically significant electrocardiogram abnormalities at screening; and moderate or severe hypertension.

For all studies, protocols were approved by ethics committees (see Supplemental Table 1 for a complete listing) and conducted in accordance with International Conference on Harmonisation Good Clinical Practice and the principles of the Declaration of Helsinki. All study participants were required to provide written, informed consent before entering the studies.

### Measures

The Y-BOCS-BE [1] is a 10-item clinician-rated scale (0?=?no symptoms to 4?=?extreme symptoms); total score ranges from 0 to 40. The items assess the obsessiveness of BE thoughts (time occupied by thoughts about BE; interference due to thoughts about BE; distress associated with thoughts about BE; resistance of thoughts about BE; degree of control over thoughts about BE) and BE behaviors (time spent on compulsive behaviors about BE; interference due to BE; distress associated with BE; resistance to BE; degree of control over BE). In these studies, the Y-BOCS-BE was assessed at baseline and weeks 4, 8, and 12/early termination (ET) in the short-term efficacy studies [6] and at open-label baseline and weeks 4, 12 (randomized-withdrawal baseline [RWB]), 16, 20, 24, 28, 32, 36, and 38/ET in the maintenance-of-efficacy study [8].

Several validated instruments were used as reference measures. The 16-item self-report BES [9] assesses behavioral, affective, and attitudinal components of the subjective BE experience; impulsivity and compulsivity are independently assessed. The BES was assessed at baseline and weeks 4, 8, and 12/ET in the short-term efficacy studies. The 51-item self-reported TFEQ [10] assesses three dimensions of eating behavior: cognitive restraint, perceived hunger, and emotionally based disinhibition of eating. The TFEQ was assessed at baseline and weeks 4, 8, and 12 in the short-term efficacy studies. The CGI-S and Clinical Global Impressions-Improvement (CGI-I) scales [11] evaluate the severity of BED symptoms and symptom improvement over time. The CGI-S was rated from 1 (normal, not at all ill) to 7 (among the most extremely ill); the CGI-I was rated from 1 (very much improved) to 7 (very much worse). In all studies, the CGI-S was assessed at all study visits and the CGI-I was assessed at all post-baseline visits. Study participants also recorded BE in a daily diary that was collected at each study visit.

### Psychometric evaluations

Psychometric modeling was conducted using Mplus^®^ version 8 for exploratory factor analysis (EFA) estimation and flexMIRT^®^ version 3 (Vector Psychometric Group, Chapel Hill, NC) for estimation of item response theory (IRT) models. Estimates of reliability, validity, meaningful improvement, and treatment efficacy were conducted with SAS^®^ version 9.4 (SAS Institute Inc., Cary, NC). There were no missing data at baseline where psychometric models were estimated. Therefore, no missing data handling methods were required or applied to any of the latent variable measurement models used in this study. The maximum proportion of missing data post baseline occurred at week 4, but was less than 1% (3 of 313 subjects, or 0.96%). Therefore, consistent with the trial justification that such negligible missing data rates are largely inconsequential [12], no missing data techniques were used beyond complete case.

#### Descriptive Y-BOCS-BE evaluations

Item response distributions were evaluated by examining the response frequencies for each item at baseline: the sparseness of response option endorsement and floor and ceiling effects (operationally defined as a threshold of 20% of participants endorsing the lowest or highest response option). Inter-item polychoric correlations were estimated at baseline across all items to evaluate provisional domain patterns and identify item redundancies.

#### Y-BOCS-BE domain structure

Although the original validation study examined the domain structure of the Y-BOCS-BE [1], the domain structure was re-examined in this manuscript using the two short-term efficacy studies of LDX described above. Domain specification followed a two-stage procedure in which evidence obtained from both EFA and IRT was used to arrive at an empirically justifiable domain solution. Selection of the final domain solution was based upon balancing model fit, clinical input, interpretability of the solution, and scoring statistics [13]. To control for the risk that the two-stage procedure might arrive at a solution due either to over-fitting or capitalization on sample-specific characteristics [14], the solution obtained from study 1 was independently replicated in study 2. The reproducibility of the solution was compared between the two studies to determine whether the solution was stable [15]. Model fit indices for EFA model selection were obtained from limited information estimation. Specifically, the mean and variance adjusted weighted least squares (WLSMV) estimator was used. Reported EFA item parameter estimates, item response theory (IRT) model fit, and IRT item parameters were all obtained from full-information marginal maximum likelihood estimation. Details of the modern psychometric methods are given in the Supplemental Materials. Two competing domain models were examined. One model was a MIRT with domains equal to those obtained from the final EFA solution, and the other was a bifactor model [16], which used the same domain structure as the MIRT augmented by a general domain reflecting overall BED severity.

After agreement on the interpretability and clinical relevance of the best fitting solution was achieved, scoring statistics were computed at baseline to determine how best to use the Y-BOCS-BE to characterize binge eating severity. Scoring statistics computed at baseline compared whether domain scoring (based on the MIRT) or a total score (based on the bifactor) was more appropriate for characterizing binge eating severity. Scoring statistics employed included the *?*, *?*_H_, the ratio of *?*_H_ to *?*, the explained common variance (ECV), and the *H* statistic [13].

#### Y-BOCS-BE scoring, reliability, validity

Reliability, validity, and meaningful change were estimated for Y-BOCS-BE sum scores in studies 1 and 2. Internal consistency was assessed at baseline using Cronbach?s *?*, with estimates ??0.7 considered acceptable. Test?retest reliability was estimated via intra-class correlation coefficients with two retest intervals: baseline to week 4 and week 4 to week 12 conditioned upon no change on anchor variables (CGI-I and CGI-S). Concurrent validity was estimated via Spearman correlations at baseline, week 4, and week 12 between Y-BOCS-BE scores and binge days, TFEQ domains (cognitive, disinhibition, and hunger), and BES total score.

#### Y-BOCS-BE clinically meaningful improvement and efficacy

Meaningful change was computed on Y-BOCS-BE change scores computed between baseline and week 12 using both distribution- and anchor-based estimates of minimal clinically important improvement (MCII). Distribution-based estimates included 1 and 0.5 baseline standard deviation (SD) and baseline standard error of the measurement (SEM). In addition, following Harvill 1991 [17], the baseline SD and SEM were used to index the amount an individual needed to improve beyond the average change score (?*?*) to reflect meaningful change. These difference estimates are noted as ?*?*?Baseline SD and ?*?*?Baseline SEM, respectively. Anchor-based MCII was estimated by comparing Y-BOCS-BE change score point and interval estimates for minimal improvement defined by the CGI-S (1-point improvement between baseline and week 12) and CGI-I (response category 3, with descriptor of ?minimally improved?). In addition to point estimates, cumulative distribution functions (CDFs) stratifying on CGI-I and CGI-S were plotted; separation of CDFs at the meaningful change estimate location between treatment arms was tested.

Mixed-effects models for repeated measures (MMRM) were used to assess treatment efficacy using Y-BOCS-BE change from baseline scores based on the full analysis set (FAS), as defined for each study [6, 8]. An unstructured error covariance matrix was used for the repeated measures. Efficacy estimates were based on least squares (LS) mean contrasts at each post-baseline visit. The MMRM was adjusted for baseline Y-BOCS-BE scores and the baseline Y-BOCS-BE score by treatment interaction.

For the maintenance-of-efficacy study (study 3), efficacy analyses differed slightly owing to the randomized-withdrawal study design. Y-BOCS-BE scores obtained during the randomized-withdrawal period were modeled as outcomes in a linear mixed model, with the primary predictors of time, treatment, and treatment by time. Covariate adjustments included the RWB Y-BOCS-BE scores and time by RWB score interaction. An unstructured residual covariance matrix was used in this model.

## Results

### Participant demographics and disposition

Participant disposition and demographics have been described in detail elsewhere [6, 8]. In brief, the FAS included the following number of participants: study 1, *n*?=?374 (LDX, *n*?=?190; placebo, *n*?=?184); study 2, *n*?=?350 (LDX, *n*?=?174; placebo, *n*?=?176); and study 3, *n*?=?267 (LDX, *n*?=?136; placebo, *n*?=?131). Across studies, most participants were white (74.0?84.3%) and were women (86.3?87.6%); mean?±?SD age and BMI, respectively, ranged from 38.0?±?10.04 to 38.7?±?10.03 years and 33.43?±?6.245 to 33.89?±?6.050 kg/m^2^. Mean?±?SD duration of exposure (in days) was 75.7?±?20.81 with LDX and 76.6?±?20.72 with placebo for study 1; 75.8?±?20.14 with LDX and 73.1?±?22.99 with placebo for study 2; and 157.6?±?51.60 with LDX and 98.9?±?72.83 with placebo during the randomized-withdrawal phase of study 3.

### Psychometric evaluations of the Y-BOCS-BE

#### Descriptive evaluations

The missing data rate was negligible (baseline, 0%; week 4, 0.96%; week 12, 0.32%). Item distribution analyses indicated there were no floor or ceiling effects. Inter-item correlations were weak at baseline (range, 0.0?0.70), with only two correlations exceeding 0.6, and were acceptable at week 12 (range, 0.62?0.88).

#### Dimensionality assessments

Model fit indices indicated that the Y-BOCS-BE domain structure was optimally characterized with a three-factor model interpreted after an oblique quartimax rotation (*?*^2^*P* value?<?0.0001; RMSEA [95% CI]?=?0.09 [0.07?0.11]; CFI?=?0.98; TLI?=?0.94; SRMR?=?0.04); this structure was carried forward into the IRT model estimation. The three domains of obsessive/compulsive, restraint, and control were defined, respectively, by time spent on, disruption of life due to, and distress associated with BE thoughts and actions (items 1?3, 6?8); ability to resist BE thoughts and actions (items 4 and 9); and ability to control BE thoughts and actions (items 5 and 10). These domains were modestly correlated (*r*?=?0.24, 0.22, 0.32, respectively).

Because this three-domain structure differed from both the two- and seven-domain solutions reported by Deal et al. [1], confirmatory analyses were performed to assess whether the three-domain structure fits better than the parsimonious two-domain structure reported by Deal et al. [1]. These structures were compared to a bifactor modification to the three-domain structure, which was found to achieve the best fit across all indices (C2 *P* value?=?0.0001; RMSEA [95% CI]?=?0.09 [0.07?0.11]; AIC?=?8394.36; BIC?=?8630.14) and minimize local dependence. The bifactor solution, emphasizing measurement of BE severity in a total domain, was clinically determined to also possess the greatest verisimilitude and interpretability. Consequently, the bifactor structure was retained in the IRT model estimation. A full explication of the evidence supporting this conclusion is contained in the Supplemental Materials.

**Fig. 1:**
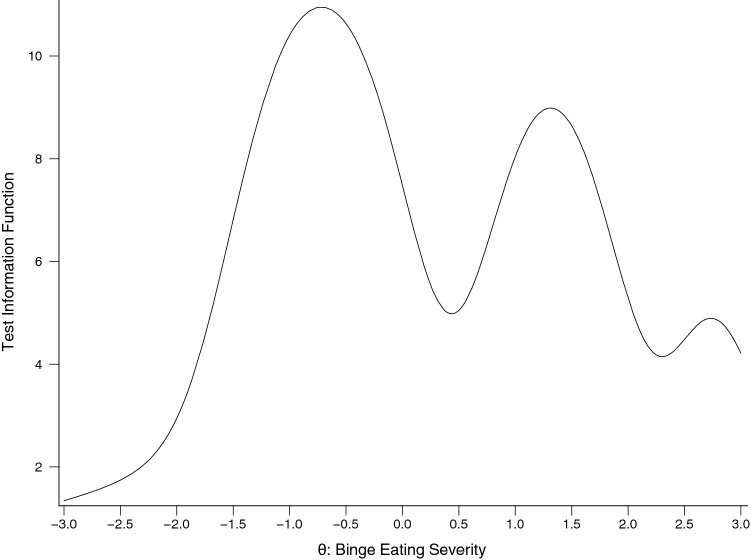
Final IRT model TIF solution. *IRT* item response theory, *TIF* test information function, *Ø* latent binge eating severity

Figure 1 depicts the test information function (TIF; proportional to scale reliability) for the final IRT model solution. The TIF is multimodal, with score reliability maximized at approximately 1 SD above and below the mean, which is identified by 0 on the x-axis, for the latent BE severity scale.

#### Scoring, reliability, and validity

Baseline scoring statistics supported the use of total score (*?*?=?0.94; *?*_H_?=?0.89; *?*_H_/*?*?=?0.95; ECV?=?0.95; and H?=?0.87). Definitions and interpretations of these statistics are given in Rodriguez et al. [13]. Descriptive statistics characterizing the distributions of these scores for baseline, week 4, and week 12 are presented in Table 1, and reflect the substantial reductions in post-baseline scores.

**Table 1:** Y-BOCS-BE score descriptive statistics short-term efficacy studies stratified by visit

Visit	*n*	Mean	SD	Minimum value	Maximum value
Baseline	313	21.7	4.9	9	39
Week 4	310	10.3	8.3	0	31
Week 12	313	9.5	8.2	0	33

*SD* standard deviation, *Y*-*BOCS*-*BE* Yale-Brown Obsessive Compulsive Scale modified for Binge Eating

Table 2 summarizes internal consistency reliability at baseline and week 12. Y-BOCS-BE item to total correlations and item *?* were lower at baseline for each item (ranges: correlations, 0.20?0.56; Cronbach?s *?*, 0.74?0.78) and for total score (Cronbach?s *?*, 0.77) than at week 12 for each item (ranges: correlations, 0.74?0.85; Cronbach?s *?*, all 0.95) and total score (Cronbach?s *?*, 0.96). However, baseline total score internal consistency achieved the pre-specified acceptability criterion.

**Table 2:** Y-BOCS-BE internal consistency estimates at baseline and week 12

	Item:total correlation		Item-level Cronbach?s *?*		Y-BOCS-BE total score Cronbach?s *?*	
	Baseline	Week 12	Baseline	Week 12	Baseline	Week 12
Y-BOCS-BE Item 1: binge thought time	0.48	0.81	0.75	0.95	NA	NA
Y-BOCS-BE Item 2: binge thought disruption	0.56	0.76	0.74	0.95	NA	NA
Y-BOCS-BE Item 3: binge thought distress	0.52	0.81	0.74	0.95	NA	NA
Y-BOCS-BE Item 4: resist thoughts	0.42	0.76	0.76	0.95	NA	NA
Y-BOCS-BE Item 5: control thoughts	0.46	0.84	0.75	0.95	NA	NA
Y-BOCS-BE Item 6: binge time	0.38	0.82	0.76	0.95	NA	NA
Y-BOCS-BE Item 7: binging disrupt	0.53	0.80	0.74	0.95	NA	NA
Y-BOCS-BE Item 8: binging distress	0.47	0.85	0.75	0.95	NA	NA
Y-BOCS-BE Item 9: resist binge	0.20	0.74	0.78	0.95	NA	NA
Y-BOCS-BE Item 10: control binge	0.35	0.85	0.77	0.95	NA	NA
Y-BOCS-BE total score	NA	NA	NA	NA	0.77	0.96

*NA* not applicable, *Y*-*BOCS*-*BE* Yale-Brown Obsessive Compulsive Scale modified for Binge Eating

Test?retest reliability correlations between baseline and week 4 in participants whose CGI-S ratings did not change between assessments or who had CGI-I ratings of ?no change? between assessments were low (*r*?=?0.34 and *r*?=?0.59). Sensitivity analyses of test?retest reliability correlations between week 4 and week 12, conditioned on similar CGI-S and CGI-I anchors, were acceptably higher (*r*?=?0.77 and *r*?=?0.90).

At baseline, convergent validity estimates were weak (*r*???0.4) across all validators (Table 2). At week 4, convergent validity estimates increased for the TFEQ disinhibition and hunger domains but remained low for binge days and the TFEQ cognitive domain (Table 3). Convergent validity estimates were high for all validators (Table 3; all *r*???0.66) at week 12 except for binge days and the TFEQ cognitive domain.

**Table 3:** Convergent validity estimates (Spearman correlations) with Y-BOCS-BE scores at baseline, week 4, and week 12

Validator	Baseline	Week 4^a^	Week 12
Binge days	0.24	0.08	0.01
TFEQ cognitive domain	??0.05	??0.15	??0.14
TFEQ disinhibition domain	0.11	0.60	0.72
TFEQ hunger domain	0.09	0.62	0.72
BES total score	0.27	ND	0.77

*BES* Binge Eating Scale, *ND* not determined, *TFEQ* Three-Factor Eating Questionnaire, *Y*-*BOCS*-*BE* Yale-Brown Obsessive Compulsive Scale modified for Binge Eating

^a^Analyses not conducted on week 4 data for the BES

The discussion section contains an extensive clinical explanation of the relationship between poor BED insight and self-monitoring effects, addressing the low test?retest reliability and convergent validity.

#### Clinically meaningful improvement and efficacy

Distribution-based MCII estimates for ?*?*?Baseline SD and ?*?*?Baseline SEM, respectively, were ?17.04 and ?14.48 in study 1 and ?15.06 and ?12.67 in study 2. Anchor-based score estimates for ?minimal improvement? on the CGI-I or a 1-point improvement on the CGI-S from baseline to week 12, respectively, were ?5.00 and ?6.78 in study 1 and ?4.52 and ?5.63 in study 2. The proportions of participants achieving the anchor-based estimates were excessively high (>?80%). Therefore, the distribution-based estimates (?*?*?Baseline SD and ?*?*?Baseline SEM) reported above were retained.

**Fig. 2:**
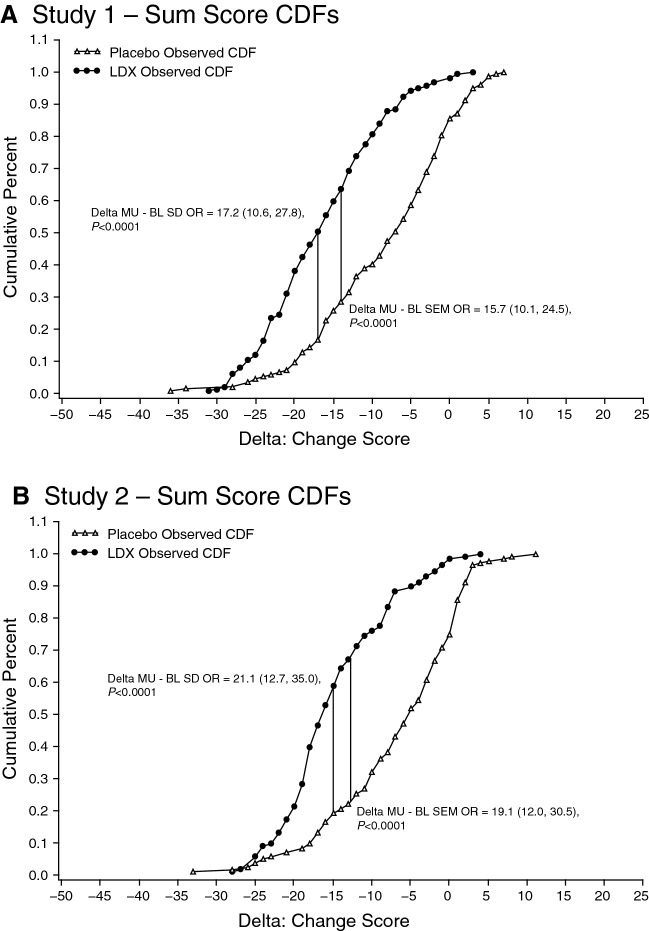
CDF curves for the Y-BOCS-BE with modeled ORs imposed at MCII locations. Panels **A** and **B**: Based on sum scores in studies 1 and 2, respectively.* BL* baseline, *CDF* cumulative distribution function, *MCII* minimal clinically important improvement, *OR* odds ratio, *SD* standard deviation, *SEM* standard error of measurement, *LDX* lisdexamfetamine dimesylate, *Y*-*BOCS*-*BE* Yale-Brown Obsessive Compulsive Scale modified for Binge Eating

Observed treatment-stratified CDF curves with estimated odds ratios corresponding to estimated MCII values are shown in Fig. 2. MCII estimates based on ?*?*?Baseline SD or ?*?*?Baseline SEM met criteria for optimal MCII for scores (Fig. 2A). Fifty percent of participants in the LDX group and 16.6% in the placebo group achieved a degree of improvement that met or exceeded the ?*?*?Baseline SD estimate; 63.5% of participants in the LDX group and 28.0% in the placebo group achieved a degree of improvement that met or exceeded the ?*?*?Baseline SEM estimate. Corresponding CDF curves for study 2 (Fig. 2B) also showed substantial separation between treatment groups.

LDX treatment effects across all studies are summarized in Table 4. The LS mean treatment difference for change from baseline (LDX vs placebo) significantly favored LDX over placebo at weeks 4, 8, and 12 (all *P?*<?0.0001) in studies 1 and 2; these findings are consistent with the primary publication [6]. The LS mean treatment difference for change from randomized-withdrawal baseline (LDX vs placebo) significantly favored LDX over placebo at weeks 16, 20, 24, 28, 32, and 38 (all *P*?<?0.0001) in study 3, which is consistent with the primary publication [8].

**Table 4:** Efficacy of LDX on the Y-BOCS-BE scores, FAS

Contrast (LDX vs placebo)	LS mean	LS mean	*P* value	Semipartial *?*^2^ (%)
	contrast	95% CI		
Study 1				
Overall			<?0.0001	29.8
Change from baseline to Wk 4	??6.53	??8.09, ??4.98	<?0.0001	9.1
Change from baseline to Wk 8	??6.94	??8.43, ??5.44	<?0.0001	11.2
Change from baseline to Wk 12	??7.40	??8.93, ??5.88	<?0.0001	12.2
Study 2				
Overall			<?0.0001	98.4
Change from baseline to Wk 4	??7.11	??8.65, ??5.56	<?0.0001	0.2
Change from baseline to Wk 8	??7.56	??9.16, ??5.96	<?0.0001	0.2
Change from baseline to Wk 12	??7.95	??9.53, ??6.36	<?0.0001	0.3
Study 3				
Overall			<?0.0001	29.3
Change from RWB to Wk 16	??5.07	??6.57, ??3.57	<?0.0001	12.4
Change from RWB to Wk 20	??6.40	??8.14, ??4.65	<?0.0001	14.9
Change from RWB to Wk 24	??5.16	??6.61, ??3.72	<?0.0001	14.2
Change from RWB to Wk 28	??6.15	??7.66, ??4.64	<?0.0001	18.5
Change from RWB to Wk 32	??4.42	??5.84, ??3.00	<?0.0001	10.7
Change from RWB to Wk 38	??5.58	??7.25, ??3.90	<?0.0001	12.3

*FAS* full analysis set, *LDX* lisdexamfetamine dimesylate, *LS* least squares, *RWB* randomized-withdrawal baseline (Wk 12), *Wk* week, *Y*-*BOCS*-*BE* Yale-Brown Obsessive Compulsive Scale modified for Binge Eating

## Discussion

This study described the psychometric evaluation of the Y-BOCS-BE based on phase 3 clinical data from two independently conducted and identically designed short-term efficacy studies and a maintenance-of-efficacy study of LDX in adults with BED. These analyses demonstrated that the Y-BOCS-BE possesses a bifactor structure composed of a general binge eating severity domain and three subdomains (obsessive/compulsive, restraint, and control) and exhibits strong internal consistency. Test?retest reliability from baseline to week 4 and convergent validity were poor at baseline but substantially better from week 4 to week 12 for test?retest reliability and at week 12 for convergent validity, potentially due to LDX treatment effects and improved insight into BED at later points during the studies. MCII estimators based on raw metrics displayed considerable heterogeneity, which was reduced with score standardization. Estimated treatment effects were significant, consistent with published results [6, 8], and accounted for a substantial percentage of the variance in change scores.

The Y-BOCS-BE measured BED severity via items assessing BE thoughts and actions. These analyses demonstrated that the Y-BOCS-BE can be decomposed into three domains (obsessive/compulsive [6 items], restraint [2 items], and control [2 items]) rather than the hypothesized two domains (obsessiveness of binge thoughts, compulsiveness of binge actions) [1]. This is consistent with previously published findings, which also reported that a two-domain structure did not adequately describe the Y-BOCS-BE [1]. In these analyses, a bifactor solution of the three-domain structure fits the data optimally and was carried forward. Under the bifactor solution, subdomains are assumed to arise because of idiosyncratic effects, such as shared item phrasing, that are assumed to be noise and best explained by the common unidimensional domain of BED severity.

Inter-item correlations, internal consistency, test?retest reliability, and convergent validity were poor for analyses conducted at baseline but substantially improved at later time points. These findings are also consistent with those of Deal and colleagues, who reported lower inter-item correlations and convergent validity at baseline than end-of-study [1]. Studies of emotional function/processing in BED have shown high levels of alexithymia (inability to identify and describe emotions), impaired interoceptive awareness (ability to recognize and respond to emotional states and visceral sensations), and impaired emotion regulation, including low cognitive reappraisal [18, 19]. These findings suggest that an inability to identify and describe emotions and visceral states may negatively impact self-awareness of BED and its severity in individuals with BED. Therefore, it is possible that the weaker baseline psychometric properties of the Y-BOCS-BE are related to poor disease insight, including an inability to discriminate BED symptom severity. However, as the studies progressed, participants gained insight into their disorder through LDX treatment effects and the experience of evaluating their symptoms.

Consistent with a previous Y-BOCS-BE validation [1], MCII estimates varied within estimator across scores and within scores across estimators so meaningful agreement on a representative meaningful change estimate could not be established in either short-term efficacy study. The MCII estimates associated with the most reasonable achievement rates (50% and 63.5%) were obtained from the ?*?*?Baseline SD and ?*?*?Baseline SEM estimators, respectively. Therefore, the estimates in score reductions of 12 to 17 points were taken to represent the best estimates of clinically meaningful improvement. This estimate range is narrower than the 4- to 17-point range reported by Deal and colleagues [1]. While a meaningful change estimate of up to 17 points is large relative in regard to the potential change score range (±?40), in fact, this reflects only 21% of the total range. Moreover, given the large treatment effect of LDX, lesser meaningful change estimates produced achievement rates of???80%.

Examination of CDF curves for all scores displayed substantial separation between LDX and placebo, with estimated treatment effects being significant in all three studies and consistent with previous publications [6, 8]. The observed treatment effects accounted for a substantial percentage of the change score variance for all scores. Based on these findings, ?*?*?Baseline SEM yielded a narrower CI range, and would be the recommended metric for defining responders in a clinical trial.

Several limitations should be considered when interpreting these data. First, including only participants with protocol-defined moderate-to-severe BED without psychiatric comorbidities limits the generalizability of these findings to the more heterogeneous general population of individuals with BED. Second, although the Eating Disorder Examination Questionnaire is considered a gold standard for assessing eating disorder symptoms, it was not used to validate the Y-BOCS-BE because it was not assessed at the baseline or post-baseline visits. Third, MCII estimators based on standardized data displayed increased homogeneity and may be advantageous in educational testing and real-world evidence contexts, for which larger sample sizes can be used to generate standardized estimates for the purposes of comparison. However, they are sample dependent and do not permit individual patient benchmarking and thus may be impractical in clinical practice due to the need to focus on patient-centric and individualized assessment in the clinical practice setting. Last, when considering treatment effects in the maintenance-of-efficacy study, it is a limitation that the analysis only included participants who did not relapse during the randomized-withdrawal phase.

These analyses from large placebo-controlled studies of LDX indicate that the Y-BOCS-BE is a valuable tool for assessing BED symptoms. These analyses demonstrated that the Y-BOCS-BE can be decomposed into three distinct domains (obsessive/compulsive, restraint, and control) rather than the previously hypothesized two domains of the Y-BOCS-BE. However, a bifactor solution of the three-domain structure best fits the data and our findings were consistent with the local dependence findings contained in the supplemental material of Deal et al. [1]. Since the psychometric properties of the Y-BOCS-BE were substantially stronger at later study time points than at baseline, it may be valuable for clinicians to implement regular self-reports of BE thoughts and behaviors into their clinical practices in order to enhance patient insight into BED and possibly improve long-term outcomes. Our findings based on MCII estimators for Y-BOCS-BE total score and standardized scores set the stage for normalizing the Y-BOCS-BE and increasing the understanding of the clinical significance of Y-BOCS-BE scores and score changes to be useful both for clinical practice and clinical research.

## Electronic supplementary material

Below is the link to the electronic supplementary material.

Supplementary material 1 (DOC 180 kb)
