# Supplementary material for: A psychometric analysis and revalidation of the Yale-Brown Obsessive Compulsive Scale modified for Binge Eating in adults with binge eating disorder
Source: Qual Life Res. 2019 Aug 31;28(12):3385–94. doi: 10.1007/s11136-019-02277-8 (PMC6863951; doi:10.1007/s11136-019-02277-8)
Supplement: Supplementary file 1 — Supplementary material 1 (DOC 180 kb) [file 11136_2019_2277_MOESM1_ESM.doc]

**Supplemental Materials**

Supplemental Table 1. Listing of Institutional Review Boards/Ethics Committees by Study

| **Study 1 (ClinicalTrials.gov registry number – NCT01718483)** |
| --- |
| CEIC H. Univ de La Princesa Dña. Cecilia López García  FIB H. Univ. de La Princesa (1ª planta)  Calle de Diego León, 62  Madrid, Spain 28006 |
| Copernicus Group IRB  1 Triangle Drive, Suite 100  Durham, NC 27713, USA |
| Ethikkommission bei der Sächsischen  Landesärztekammer  Schützenhöhe 16  Dresden, Germany 01099 |
| Ethikkommission zur Beurteilung medizinischer  Forschung am Menschen  Berliner Allee 20  Hannover, Germany 30175 |
| Independent Ethics Committeee of Hospital General  Universitario GREGORIO MARAÑÓN  C/ Dr. Esquerdo 46, Pabellón de Gobierno, Planta baja  Madrid, Spain 28007 |
| Landesamt for Gesundheit und Soziales  Geschäftsstelle der Ethik-Kommission des Landes  Berlin  Fehrbelliner Platz 1  Berlin, Germany 10707 |
| McLean Hospital  115 Mill Street  Belmont, MA 02478, USA |
| National Ethics Committee  48 Av. Santescu Street, district 1  Bucharest, Romania |
| The Regional Ethical Review Board in Stockholm  Nobels väg 9, Floor D3  Stockholm, Sweden 171 65 SOLNA |
| Tajemník EK  Etická komise IKEM a TN  Vídeňská 800  Prague 4- Krč, Czech Republic 140 59 |
| **Study 2 (ClinicalTrials.gov registry number – NCT01718509)** |
| CEIC H. Univ de la Santa Creu i Sant Pau  Av. Sant Antoni Mª Claret, 167  Barcelona, Spain 08025 |
| Comitato Etico Indipendente Presso la Fondazione  PTV Policlinico Tor Vergata di Roma  Viale Oxford 81  Roma, Italy 00133 |
| Comitato per la Sperimentazione Clinica dei  Medicinali dell’ Azienda Ospedaliero Universitaria  Pisana di Pisa  Via Roma 67  Pisa, Italy 56126 |
| Copernicus Group IRB  1 Triangle Drive, Suite 100  Durham, NC 27709, USA |
| Ethik-Kommission an der Medizinischen Fakultät der  Universität Rostock  St.-Georg-Str. 108  Rostock, Germany 18055 |
| Ethikkommission zur Beurteilung Medizinischer  Forschung am Menschen  Berliner Allee 20  Hannover, Germany 30175 |
| Human Research Protection Program  D-528 Mayo Memorial Building  420 Delaware Street SE  Minneapolis, MN 55455 USA |
| Independent Ethics Committee of Hospital General  Universitario GREGORIO MARAÑÓN  C/ Dr. Esquerdo 46, Pabellón de Gobierno, Planta baja  Madrid, Spain 28007 |
| Landesamt für Gesundheit und Soziales  Geschäftsstelle der Ethik-Kommission  des Landes Berlin  Fehrbelliner Platz 1  Berlin, Germany 10707 |
| McLean Hospital IRB  115 Mill Street  Belmont, MA 02478, USA |
| National Ethics Committee  48 Av. Santescu Street, District 1  Bucharest, Romania |
| University of Cincinnati IRB  University Hall, Suite 300  51 Goodman Drive, P.O. Box 210567  Cincinnati, OH 45221, USA |
| **Study 3 (ClinicalTrials.gov registry number – NCT02009163)** |
| Western Institutional Review Board  1019 39th Avenue SE, Suite 120  Puyallup, WA 98374-2115 |
| Copernicus Group Independent Review Board  One Triangle Drive  Suite 100  PO Box 110605  Research Triangle Park, NC 27709 |
| McLean Hospital IRB  115 Mill St.  Belmont, MA 02478 |
| Partners Human Research Committee  116 Huntington Avenue, Suite 1002  Boston, MA 02116 |
| University of Cincinnati IRB  University Hall, Suite 300  Cincinnati, OH 45221 |
| CEIC Hospital Universitario de La Princesa  FIB H. Univ. de La Princesa (1ª planta)  Calle de Diego León, 62  Madrid, Spain 28006 |
| CEIC Hospital Universitario de la Santa Creu i Sant  Pau  Av. Sant Antoni Mª Claret, 167  Barcelona, Spain 08025 |
| CEIC Hospital General Universitario GREGORIO  MARAÑÓN  C/ Dr. Esquerdo 46, Pabellón de Gobierno, Planta baja  Madrid, Spain 28007 |
| Ethik-Kommission an der Medizinischen Fakultät der  Universität Rostock  St.-Georg-Str. 108  Rostock, Germany, 18055 |
| Ethikkommission bei der Sächsischen  Landesärztekammer  Schützenhöhe 16  Dresden, Germany, 01099 |
| Ethikkommission zur Beurteilung medizinischer  Forschung am Menschen bei der Ärztekammer  Niedersachsen  Berliner Allee 20  Hannover, Germany, 30175 |
| Landesamt für Gesundheit und Soziales  Geschäftsstelle der Ethik-Kommission des Landes  Berlin  Fehrbelliner Platz 1  Berlin, Germany, 10707 |
| The Regional Ethical Review Board in Stockholm  Nobels väg 9, Floor D3  Stockholm, Sweden 171 65 SOLNA |

**Domain Specification Methods**

Model selection for the EFA was based on fit indices estimated in MPlus® from mean and variance adjusted weighted least squares solutions (WLSMV). Fit indices considered included the χ^2^ test of exact fit, Root Mean Square Error of Approximation (RMSEA, <0.1 to ≥0.05, acceptable fit; <0.05 to >0, strong fit; 0, perfect fit), the Comparative Fit Index (CFI, 0 indicates poor fit; 1 indicates perfect fit), the Tucker-Lewis Index (TLI, 0 indicates poor fit; 1 indicates perfect fit), and the standardized root mean square residual (SRMR, 0 indicates perfect fit, and values <0.08 indicate good fit). Final EFA solutions were estimated via full-information graded-response exploratory item factor analysis. The final EFA solutions were rotated via oblique quartimax to maximize interpretation of loadings.

Item response theory (IRT) models were estimated at baseline to assess relationships between items and domains, refine domain specification, and define empirical scoring statistics; graded response models [1] were employed. Model fit and local dependence statistics were used to refine the optimal IRT domain structure for scoring. These indices included the C2-based χ^2^ test of exact fit and C2-based RMSEA [2], which are members of the M2 family of fit indices [3] that are appropriate for cases of small numbers of items with many response categories, as is true for the Y-BOCS-BE. In addition, model fit was evaluated with the Bayesian information criterion (BIC, smaller values are better), and Akaike’s information criterion (AIC, smaller values are better), though information criteria were deemphasized relative to the C2-based fit indices. Competing models considered included a unidimensional IRT, multidimensional IRT, and bifactor model.

**EFA Model Selection and Loading Matrix**

Model fit supporting the selection of the optimal number of factors is presented in **Supplemental Table 2**. The 3-factor model maximized fit while balancing model complexity. This model had the smallest root mean square error of approximation (RMSEA; 0.09) with lower 95% confidence limit approaching the desirable 0.05 threshold indicating close fit, and the largest Comparative Fit Index (CFI) and Tucker-Lewis Index (TLI), 0.98 and 0.94 respectively, and smallest standardized root mean square residual (SRMR; 0.04). Based on the fit indices the 3-factor model was retained to evaluate the relationship of items in the Yale-Brown Obsessive Compulsive Scale modified for Binge Eating (YBOCS-BE). The oblique quartimax rotated full-information graded-response exploratory item factor analysis loadings are presented in **Supplemental Table 3**. This solution clearly demonstrates the decomposition of items into domains influenced by shared item content. Items measuring obsessive-compulsive (binge thoughts and binge action) defined what was called the obs/comp domain. Items measuring restraint of binge thoughts or actions defined the restraint domain, and items measuring control of binge thoughts or actions defined the control domain. Inter-factor correlations indicated that the obs/comp domain correlated with the restraint and control domains 0.24 and 0.22, respectively, while restraint and control correlated 0.32 with each other.

*Supplemental Table 2. Baseline WLSMV Limited Information EFA Fit Indices*

| **Factor solution** | **χ^2^** | **DF** | ***P* Value** | **RMSEA** | **RMSEA 95% CI** | **CFI** | **TLI** | **SRMR** |
| --- | --- | --- | --- | --- | --- | --- | --- | --- |
| 1-factor | 591.84 | 35 | <0.0001 | 0.21 | 0.19, 0.22 | 0.73 | 0.66 | 0.12 |
| 2-factor | 150.08 | 26 | <0.0001 | 0.11 | 0.10, 0.13 | 0.94 | 0.90 | 0.05 |
| 3-factor | 71.31 | 18 | <0.0001 | 0.09 | 0.07, 0.11 | 0.98 | 0.94 | 0.04 |
| CFI=Comparative Fit Index; CI=confidence interval; DF=degrees of freedom; EFA=exploratory factor analysis; RMSEA=root mean square error of approximation; SRMR=standardized root mean square residual.TLI= Tucker-Lewis Index; WLSMV= mean and variance adjusted weight least squares solutions.  Interpretation of fit indices:   1. χ^2^: Non-significant *P* value indicates perfect fit, test is overpowered, thus small samples enable achievement of this criterion. 2. RMSEA: Values less than 0.1 indicate acceptable fit, values less than 0.05 indicate strong fit, values of 0 indicate perfect fit. 3. CFI, TLI: greater than 0.9 indicates acceptable fit, 1 indicates perfect fit. 4. SRMR: 0 indicates perfect fit. | | | | | | | | |

Supplemental Table 3. Baseline Full-Information Graded-Response Exploratory Item Factor Analysis Solution

| **YBOCS item** | **Item stem** | **Factor 1 loading** | **Factor 2**  **loading** | **Factor 3**  **loading** |
| --- | --- | --- | --- | --- |
| YBOCS1 | Binge thought time | 0.56 | 0.04 | 0.07 |
| YBOCS2 | Binge thought disrupt | 0.87 | –0.01 | –0.04 |
| YBOCS3 | Binge thought distress | 0.78 | –0.02 | –0.03 |
| YBOCS6 | Binging time | 0.42 | 0.14 | –0.04 |
| YBOCS7 | Binging disrupt | 0.75 | 0.03 | 0.01 |
| YBOCS8 | Binging distress | 0.53 | –0.08 | 0.17 |
| YBOCS4 | Resist thoughts | 0.01 | 1.00 | –0.03 |
| YBOCS9 | Resist binge | –0.11 | 0.55 | 0.21 |
| YBOCS5 | Control thoughts | 0.20 | 0.32 | 0.35 |
| YBOCS10 | Control binge | 0.00 | –0.01 | 1.00 |

# **IRT Model Selection and Item Parameter Estimates**

Model fit indices are presented in **Supplemental Table 4**. Neither the unidimensional nor the Y-BOCS-BE obs/comp multidimensional item response theory (MIRT) model fit the data well. Both the exploratory factor analysis (EFA) domain MIRT and the bifactor (which added a general domain to the EFA domain structure) fit the data equally well. However, the bifactor model shrunk Chen’s standardized local dependence (LD) statistic for the restraint and control items more than the other models. For the restraint domain items Chen’s standardized LD shrunk from 33.7 to 5.5 between the unidimensional model and the bifactor while the control domain items shrunk from 10.9 to 0.6, where values of 2 or below are desired.

The final bifactor item-response theory (IRT) model solution is presented in **Supplemental Table 5**. The intercepts (C1-C4) and slopes were well distributed for patient-reported outcome data, with the resist thoughts and control thoughts items demonstrating some persistent residual associations in their slopes of 3+.

Supplemental Table 4. Baseline Full Information Graded Response IRT Model Fit Indices

| **Fit statistic** | **Unidimensional** | **Obs/Comp MIRT**  **(Deal et al^.^**^a^**)** | **EFA-based**  **MIRT** | **Bifactor** |
| --- | --- | --- | --- | --- |
| C2-based χ^2^ | 336.42 | 335.33 | 123.47 | 103.08 |
| C2-based χ^2^ DF | 35 | 34 | 32 | 25 |
| C2-based χ^2^ *P* value | 0.0001 | 0.0001 | 0.0001 | 0.0001 |
| C2-based RMSEA | 0.15 | 0.15 | 0.09 | 0.09 |
| C2-based RMSEA 95%CI | 0.14, 0.17 | 0.14, 0.17 | 0.07, 0.10 | 0.07, 0.11 |
| AIC | 8608.57 | 8607.50 | 8391.38 | 8394.36 |
| BIC | 8805.05 | 8807.91 | 8599.65 | 8630.14 |

AIC=Akaike’s information criterion; BIC=Bayesian information criterion; CI=confidence interval; DF=degrees of freedom; EFA=exploratory factor analysis; IRT=item response theory; MIRT= multidimensional item response theory; RMSEA=root mean square error of approximation.

^a^Deal, L. S., Wirth, R. J., Gasior, M., Herman, B. K., McElroy, S. L. (2015). Validation of the Yale-Brown Obsessive Compulsive Scale modified for Binge Eating. *International Journal of Eating Disorders*, 48(7), 994-1004.

Supplemental Table 5. Baseline Bifactor IRT Item Parameter Solution

|  |  | **Domain-specific Slope** | | | |  | **Item intercepts** | | | |
| --- | --- | --- | --- | --- | --- | --- | --- | --- | --- | --- |
| **Item** | **Item stem** | **General** | **Obs/Comp** | **Restraint** | **Control** |  | **C1** | **C2** | **C3** | **C4** |
| 1 | Binge thought time | 0.80 | 1.04 | 0 | 0 |  | 5.02 | 2.87 | –0.52 | –3.24 |
| 2 | Binge thought disrupt | 1.22 | 3.08 | 0 | 0 |  | 3.99 | 0.62 | –4.29 | –9.43 |
| 3 | Binge thought distress | 0.93 | 1.81 | 0 | 0 |  | 4.15 | 2.11 | –1.1 | –5.02 |
| 6 | Binging time | 0.60 | 0.68 | 0 | 0 |  | 6.29 | 1.71 | –1.75 | –5.28 |
| 7 | Binging disrupt | 0.96 | 1.91 | 0 | 0 |  | 3.39 | 0.82 | –2.8 | –7.61 |
| 8 | Binging distress | 0.77 | 0.89 | 0 | 0 |  | 3.84 | 1.77 | –0.98 | –4.4 |
| 4 | Resist thoughts | 3.27 | 0 | 4.32 | 0 |  | 9.31 | 3.93 | –2.74 | –7.37 |
| 9 | Resist binge | 0.78 | 0 | 1.11 | 0 |  | 4.14 | 1.78 | –0.55 | –2.54 |
| 5 | Control thoughts | 3.05 | 0 | 0 | 1.20 |  | 7.51 | 5.45 | 1.06 | –4.12 |
| 10 | Control binge | 1.20 | 0 | 0 | 0.34 |  | 6.61 | 4.97 | 1.18 | –2.15 |

IRT=item response theory.

# **Scoring statistics**

Scoring statistics were computed at baseline to determine how best to use the Y-BOCS-BE to characterize binge eating severity. Scoring statistics employed included the ω, ω_H_, the ratio of ω_H_ to ω, the explained common variance (ECV), and the H statistic. Details of these statistics and their interpretation are given by Rodriguez et al (*Psychological Methods* 2016; 21[2], 137-150). The ω statistics decompose the internal consistency attributed to all domains (ω), a total domain (ω_H_), and the percentage of a total domain relative to all domains (ω_H_/ω). The ECV can be thought of as the proportion of variance of the item responses that can be attributed to the total domain. It is a measure of the relative strength and useful contribution of the subdomains. H is a measure of factor determinacy and quantifies the correlation between the latent domain(s) and corresponding observed domain scores. Low values of H indicate that the latent domain(s) may not be reliably specified, leading to a low likelihood that observed scores will reproducibly reflect the latent domains of intent. For all scoring statistics described, values exceeding 0.8 and approaching 1 indicate the appropriateness of a total score over domain scores. Baseline scoring statistics supported the use total score (ω=0.94; ω_H_ =0.89; ω_H_ / ω =0.95; ECV=0.95, and H=0.87).

# **Reliability, Validity, Meaningful Change, and Treatment Efficacy**

Supplemental Table 6. MCII-based Meaningful Change Estimates for Y-BOCS-BE Scores

| **MCII class** | **Definition** | **Score** |
| --- | --- | --- |
| Distribution | Baseline SD | 4.91 |
| Distribution | Baseline SEM | 2.35 |
| Distribution | Δ μ - Baseline SD | –17.04 |
| Distribution | Δ μ - Baseline SEM | –14.48 |
| Distribution | Δ μ - Δ SD | –21.08 |
| Anchor | CGI-I minimal improvement* | –5.00 |
| Anchor | CGI-S minimal improvement^†^ | –6.78 |

MCII=minimal clinically important improvement; CGI-I=Clinical Global Impressions–Improvement; CGI-S=Clinical Global Impressions–Severity; SD=standard deviation; SEM=standard error of measurement; Y-BOCS-BE=Yale-Brown Obsessive Compulsive Scale modified for Binge Eating; Δ μ=average change score.

*Rating of 3.

^†^1-point improvement between baseline and week 12.

**References**

1. Samejima, F. (1969). Estimation of Latent Ability Using a Response Pattern of Graded Scores. Richmond, VA: The William Byrd Press.

2. Cai, L.& Monroe, S. (2014). A new statistic for evaluating item response theory models for ordinal data (CRESST Report 839). Los Angeles: University of California, Los Angeles, National Center for Research on Evaluation, Standards, and Student Testing (CRESST). (<http://cresst.org/publications/cresst-publication-3208/>).

3. Maydeu-Olivares, A.& Joe, H. (2006). Limited information goodness-of-fit testing in multidimensional contingency tables. Psychometrika, 71, 713-732.
